# Demonstrating the Use of Optical Fibres in Biomedical Sensing: A Collaborative Approach for Engagement and Education

**DOI:** 10.3390/s20020402

**Published:** 2020-01-10

**Authors:** Katjana Ehrlich, Helen E. Parker, Duncan K. McNicholl, Peter Reid, Mark Reynolds, Vincent Bussiere, Graham Crawford, Angela Deighan, Alice Garrett, András Kufcsák, Dominic R. Norberg, Giulia Spennati, Gregor Steele, Helen Szoor-McElhinney, Melanie Jimenez

**Affiliations:** 1 EPSRC IRC Hub in Optical Molecular Sensing & Imaging, Centre for Inflammation Research, Queen’s Medical Research Institute, University of Edinburgh, Edinburgh EH16 4TJ, UK; akufcsak@exseed.ed.ac.uk (A.K.); Dominic.Norberg@ed.ac.uk (D.R.N.); Helen.Szoor-McElhinney@ed.ac.uk (H.S.-M.); 2 Scottish Universities Physics Alliance (SUPA), Institute of Photonics and Quantum Science, Heriot-Watt University, Edinburgh EH14 4AS, UK; dkm2@hw.ac.uk; 3 College of Science and Engineering Engagement Team, King’s Buildings, University of Edinburgh, Edinburgh EH9 3BF, UK; peter.reid@ed.ac.uk (P.R.); mark.reynolds@ed.ac.uk (M.R.); 4 James Watt School of Engineering, Biomedical Engineering Division, University of Glasgow, Glasgow G12 8LT, UK; v.bussiere.1@research.gla.ac.uk (V.B.); a.garrett.1@research.gla.ac.uk (A.G.); g.spennati.1@research.gla.ac.uk (G.S.); Melanie.Jimenez@glasgow.ac.uk (M.J.); 5 Liberton High School, Edinburgh EH17 7PT, UK; graham.crawford@liberton.edin.sch.uk; 6 St. Margaret Mary’s School, Glasgow G45 9NJ, UK; gw09deighanangela@glow.ea.glasgow.sch.uk; 7 Scottish Schools Education Research Centre (SSERC), Dunfermline KY11 8UU, UK; gregor.steele@sserc.org.uk

**Keywords:** endoscopic imaging, fluorescence imaging, fiber optics, medical imaging, medical optics instrumentation, lung disease diagnostics, public understanding/outreach, high school/introduction medicine, interdisciplinary/multidisciplinary

## Abstract

This paper demonstrates how research at the intersection of physics, engineering, biology and medicine can be presented in an interactive and educational way to a non-scientific audience. Interdisciplinary research with a focus on prevalent diseases provides a relatable context that can be used to engage with the public. Respiratory diseases are significant contributors to avoidable morbidity and mortality and have a growing social and economic impact. With the aim of improving lung disease understanding, new techniques in fibre-based optical endomicroscopy have been recently developed. Here, we present a novel engagement activity that resembles a bench-to-bedside pathway. The activity comprises an inexpensive educational tool (<$70) adapted from a clinical optical endomicroscopy system and tutorials that cover state-of-the-art research. The activity was co-created by high school science teachers and researchers in a collaborative way that can be implemented into any engagement development process.

## 1. Introduction

Lung diseases are prevalent throughout the world and include a range of conditions such as asthma, acute respiratory infections, tuberculosis, lung cancer and chronic obstructive pulmonary disease (COPD). COPD affects 64 million people worldwide and results in approximately three million death per year, which makes it the third leading cause of death worldwide [1]. Lung cancer is the leading cause of cancer death worldwide (18.4 % of all cancer deaths), killing 1.7 million people per year, and this number is predicted to rise [2]. Tuberculosis (TB), which most often affects the lungs, is one of the leading causes of death from an infectious agent. In 2017, an estimated ten million people contracted TB and 1.3 million people died from the infection globally [3]. With drug-resistance on the rise, TB and other bacterial infections continue to be a major public health concern.

From X-rays to optical endomicroscopy (OEM) systems, optics have played a crucial role in the development of systems as well as understanding light–matter interactions that can help to diagnose such diseases. Optics, and especially biomedical optics, is therefore at the core of a wide range of research projects that will shape our future. As such, engagement with a wider audience, the research community, and the general public is of prime importance for influencing policy and cultural change. One way forward is to embed public engagement into the research culture through explicitly stating it in funding announcements [4]. While several formal engagement papers have been published [5,6,7,8], literature dedicated to and learning from engagement can remain hard to find. Although some examples of knowledge exchange around engagement do exist [9,10,11,12,13,14,15,16,17], activities and materials are generally not readily available for the wider research community and often not reviewed by peers. This results in long design/test/optimisation phases to develop new activities that could be minimised by further promoting knowledge exchange in the field. As one example, optical fibres present the advantage of being familiar to a non-scientific audience due to their extensive use in daily life (e.g., for telecommunication) and are consequently perfect vectors for transmitting challenging interdisciplinary concepts. Over a one-year period, we, a team of physicists and engineers, have developed in a co-production process with teachers a new tool to demonstrate the use of optical fibres in biomedical applications. To date, the tool has been deployed in 10% of Scottish secondary schools and has been presented at various venues for engagement and design optimisation. The tool is relatively low-cost (<$70), versatile, and has been adapted for use by physics teachers in classrooms and by researchers at outreach events.

The aims of this paper are to (i) propose a novel and easy-to-implement teaching session and educational tool designed to engage a wider audience with the topic of applied optical physics, and (ii) to encourage researchers to adopt or adapt the process through which this teaching session was developed for their own engagement work. Specifically, we have focused on the use of optical fibres within biomedical sciences. The educational tool, which we describe in detail, is analogous to a state-of-the-art OEM system and is linked to a diagnostic challenge around pulmonary diseases but can be easily adapted to other topics. The background know-how is presented as a short review describing current lung disease diagnosis methods and recent approaches to innovate the field that use optical fibres to perform molecular imaging and sensing in the distal lung. To complement the educational tool, an outline for tutorials and supporting materials to aid with their delivery have been provided.

## 2. Review: Optical Fibres and Lung Disease Diagnostics

### 2.1. Optical Fibres: From Telecommunications to Biomedical Imaging and Sensing

**Figure 1:**
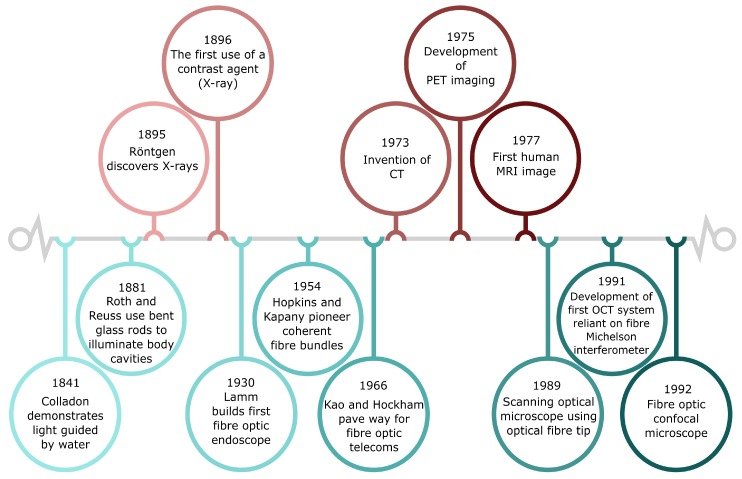
Timeline of medical imaging developments. (**Top**) Brief overview of developments in medical imaging such as CT (Computed Tomography), PET (Positron-Emission Tomography), MRI (Magnetic Resonance Imaging) and OCT (Optical Coherence Tomography). (**Bottom**) Timeline of the development of optical fibres and some applications in medical imaging.

The guiding of light through total internal reflection was first demonstrated in the 1840s by Jean-Daniel Colladon and Jacques Babinet with a ‘light-pipe’, or light fountain [18] (Figure 1). It was more than one hundred years later that light guidance through glass was established as potentially revolutionary to telecommunications in landmark work carried out in 1966 by Kao and Hockham [19], for which Kao was awarded the Nobel Prize in 2009 [20]. At the time, optical fibres suffered from rapid transmission loss and signal distortion from modal dispersion. Kao and Hockham predicted that losses of 20 dB /km at 0.6 μm were practically achievable as a result of improved glass purification and the use of single-mode fibres. Today, commercial single-mode fibres achieve <0.2 dB /km at 1.55 μm and multimode fibres achieve <8 dB /km at 0.8 μm. Further rapid advancement in technology, paired with an increased need for fast data transfer, has seen optical fibres become ubiquitous within telecommunications. Such prevalence has resulted in widely available and inexpensive fibres, which has led to their extensive employment in a wide range of scientific fields such as astronomy and the life sciences.

Imaging fibres were first crudely developed in the early 20th century to study the lining of the stomach [21]. These fibres were contained in rigid structures which made them inflexible, limiting the interest in this emergent technology. Indeed conventional endoscopes, which contain a train of lenses, were and still are the preferred technology in gastroenterology because they offer a wide viewing angle and a high light throughput. However, remote imaging and sensing in less accessible narrow cavities and tubular structures inside the human body can be achieved with flexible optical fibres. They offer great advantages for use in a clinical environment as they are immune to external electromagnetic radiation, easy to sterilise, non-toxic and bio-compatible, and therefore can be brought into direct contact with the tissue surface. Furthermore, the low-cost material makes them ideal for disposable single-use applications.

Coherent fibre bundles are made up of many thousands of cores that maintain their relative orientation throughout the length of the bundle. The pioneering work on these bundles in the 1950s by Hopkins and Kapany [22] triggered renewed interest in optical fibres within the context of biomedical imaging. Each core within the bundle acts as an individual transmitter of light from the distal end. Together, the cores form an image at the proximal end that can be viewed in real-time. In general, coherent fibre bundles are flexible and compact in size, with diameters on the order of hundreds of μms, which permits minimally invasive remote light delivery to various organs of the body [23,24,25,26]. Image resolution is increased by dense core packaging up to the limit of core crosstalk [27], a power coupling between the individual cores, which introduces blurring and reduces image contrast. Approaches to overcome this limitation include high levels of glass doping, structured packaging of varied core sizes, and signal processing [28,29]. Recently, a low index contrast imaging fibre bundle was reported using commercially available preforms typically used in the telecommunication industry which achieved low core crosstalk by relying on a square array of cores [30].

Beyond imaging, optical fibres have also been used extensively for sensing purposes. Optical fibre sensors have been explored for a wide range of applications including sensing of temperature [31,32,33], pressure [33,34], enzyme activity [35,36], presence of nucleic acids [36,37], pH [38,39], and oxygen saturation [38]. Detailed reviews can be found elsewhere [33,34,36,40,41,42]. Optical fibre sensors can be differentiated into intrinsic and extrinsic sensors. Intrinsic sensors use the fibre itself as the sensing element, whereas extrinsic sensors need an additional sensing element. Intrinsic sensors range from Fibre Bragg gratings, interferometers, resonators to distributed sensors. They allow for single or multiple point sensing or continuous monitoring along the length of the fibre. For extrinsic sensors, the optical fibre only transmits the light while the sensing element is external to it. There are a wide range of fibre probe designs available, but it is the application that defines the operational frame and most suitable parameters. These parameters might include: number of fibres, geometry of probe, core diameter, numerical aperture and fibre tip design. Optical fibre sensors have been utilised for functional spectroscopy and imaging down to the single-molecule level allowing insight into the local micro-environment of molecules, cells and tissue with a wide range of modalities, including white-light spectroscopy [43], fluorescence [24], Raman [39,44,45,46] and optical coherence tomography (OCT) [47], as well as combined modalities [48,49,50].

### 2.2. Diagnostic Challenges of Pulmonary Diseases

Globally, more than one billion people suffer from either acute or chronic respiratory conditions. Major contributory factors to the prevalence and high mortality of many respiratory diseases are non-specific initial symptoms, such as a cough, fever, shortness of breath, and chest pain, in combination with a lack of efficacious diagnosis methods. Medical imaging techniques such as X-ray, CT (computed tomography), PET (positron-emission tomography), and MRI (magnetic resonance imaging) have long existed as important tools to ‘see’ respiratory diseases, diagnose them and follow their development in patients, (Figure 1). From 1900 onwards, only five years after their discovery by Wilhelm Röntgen [51], X-rays became invaluable for imaging cancer [52] and detecting tuberculosis [53] in the lung, especially in combination with contrast agents [54]. 3D imaging by CT was developed in the 1970s [55]. However, X-ray, CT and MRI scans provide only structural and not functional information which can limit diagnostic utility. The PET tomograph was developed in 1975 and is now frequently combined with a CT or MRI to provide both anatomic and metabolic imaging [56]. PET/CT has assumed great importance within oncology due to its ability to provide metabolic information related to tumour cells [57]. However, these common diagnosis tools are costly and do not provide molecular imaging resolution. Furthermore, they are often not suitable for patients in critical care, or for recurring use, and expose the patients to ionising radiation [58].

**Figure 2:**
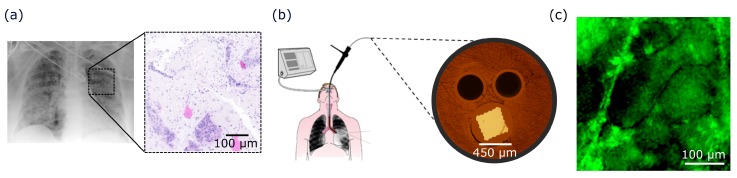
How the current clinical pathway of respiratory diagnosis could be altered with optical endomicroscopy (OEM). (**a**) Typically, respiratory symptoms in the intensive care units (ICUs) will be investigated via an X-ray. Investigations may progress further to a histological examination of excised tissue. (**b**) The fibred OEM system could provide diagnostic help in situ. The fibre device is comprised of a coherent fibre bundle (square array) to allow for imaging and two capillary channels for delivery and microlavage of fluid. (**c**) Fluorescence from tissue visualised within the alveolar space in real-time. Exogenous fluorophores can be added to label pathology and improve disease understanding. Image modified with permission from Parker et al. [63].

Although the exact clinical pathway to diagnosis is dependent on each case, accurate diagnosis of lung diseases is typically performed through invasive biopsies and lavages, where tissue and fluid samples from the lungs are taken and analysed in a histology lab (Figure 2a). Histological examination can take several days which can cause delays in the stratification of patients with suspected lung disease. In many cases, this results in poorer patient outcomes [59]. For patients with suspected infection, doctors frequently prescribe a ‘cocktail’ of antibiotics since they are unable to specify the disease-causing pathogen quickly and are unwilling to suspend treatment. Overuse of antibiotics, in the medical field and elsewhere, is a continuing and significant contributor to the global threat of antimicrobial resistance (AMR) [60,61,62]. The availability of fast and accurate diagnosis methods is a key step toward prudent antibiotic use and more efficacious treatment for patients.

OEM can be enabled with fibre bundles to provide a low-cost and minimally invasive solution for rapid and frequent measurements with high resolution at both micro- and macroscopic levels in vivo. The fibre bundles are small and flexible enough to perform microscopy of various organs of the body, to provide in situ imaging within a clinical setting (Figure 2b). Changes in the absorption, transmission and scattering of light when interacting with matter allow for differentiation between normal and abnormal tissue. In particular, fluorescence-based OEM systems have stimulated interest because fluorescence can provide information not only on the tissue architecture and composition but also on the local environment of the fluorophores (Figure 2c). This has been explored through the autofluorescence of molecules native to tissue such as co-enzymes (NADH and FAD), structural molecules (collagen and elastin), lipids and porphyrins [64]. A further approach is the use of synthesized fluorophores to interrogate molecules and their dynamics or to be used as a contrast agent between tissue and microorganisms [65,66]. The current standard for in vivo clinical fluorescent OEM is a fluorescence confocal imaging system [67,68,69] which images the lung through an optical fibre bundle by taking advantage of the strong autofluorescence mainly from elastin when excited with a laser at 470 nm [70]. The utility of this system can be enhanced through multiplexing with fluorescent molecular probes which can identify bacterial burdens [63,65,71]. Recently, a multicolour widefield fluorescence endomicroscopy system was developed which enables guidance using green tissue autofluorescence and bacterial detection with a red bacterial probe [72,73]. This system forms the basis of the education tool.

## 3. Translation from Biomedical Research to Public Engagement

The main goal of the activity developed here is to determine the colour of a sample without seeing it by using optical fibres connected to an electronic board as a sensor. This mimics detection of lung diseases: the colour of each sample is characteristic of a lung condition (e.g., red would confirm the presence of bacteria in the lungs [73]). Importantly, the activity was developed to be relevant to the curriculum taught in schools locally (herein Scotland) so that (i) researchers could easily collaborate with physics teachers and (ii) the biomedical story could be readily used by physics teachers in classrooms. Towards that goal, the tool was co-designed by researchers and teachers to address the learning needs of 12–14-year-old students and identify the most relevant teaching areas.

We established that some of the curriculum aims were unsuitable for exploration in a practical session, and used this opportunity to split our work between a set of teaching materials which provide the necessary context and a tool that provides a focus for free enquiry and student-led research. The topics that were more suited to direct interaction were used as a framework to translate more complex aspects of the OEM system into a simplified educational tool that could be constructed and used by students. The elements that were deemed essential to this were the use of fibres to access difficult-to-reach areas, an understanding of how light interacts with objects, and the use of electronic boards to control the light going through the sensor. During the development, a practical compromise had to be made by changing the tool’s operating modality from fluorescence to reflective sensing.

Based on the feedback received from partner teachers, materials available in schools sometimes limit student learning of physics to abstract ideas. However, it has been shown that placing physics within a meaningful context is crucial to the understanding of underlying scientific concepts. This is particularly important for understanding across genders equally [74]. Thus, we aimed to produce an educational tool that improved physics learning by showing links at each stage to biomedical science and public health. Although the tool was developed with the Scottish curriculum in mind, we strongly believe that this notion of meaningful contextualisation will be useful to a wider community.

Key concepts from the research were identified and curated into teaching material [75] that demonstrates these appropriately in conjunction with the tool. The teaching material introduces the concept of diagnostic uncertainty, tells the story of a generalised but realistic clinical pathway and shows how current research aims to disrupt this pathway to improve clinical outcomes. Thus, the students can engage with the tool through the use of a case study that reflects a common hospital situation. The goal is that having taken part in the activities students would: (i) understand the implications of diagnostic uncertainty; (ii) learn about the basic principles behind the experimental techniques used in an OEM system; (iii) understand how the educational tool is analogous to an OEM system; (iv) carry out work as a team, construct the tool and use it in a structured way; (v) develop skills in taking measurements, making graphs, and evaluating the final medical results including understanding the importance of calibration in experiments; and (vi) appreciate the errors that can occur in any experiment, due to misalignments in construction, component variability and measurement inaccuracies.

The production of the teaching material was guided by a number of interactions, partly drawing on the researchers’ collective experience of public engagement and the observed points of tension in other contexts. Principally, however, the level and relevance of the content was iteratively developed through close collaboration with teachers. This collaboration took the form of initial knowledge exchange events allowing researchers and teachers to create a dialogue about the key concepts and their relevance and usefulness to a school context followed by extensive opportunities for feedback on all aspects of the materials. By placing the novel research in the context of the teachers’ experiences, a common ground was established that allowed for the production of tailored and pedagogically relevant materials. The material is intended to be delivered over three separate science lessons, although it is easily adaptable to suit the needs of the teacher. As an example, the tool was also tested in our partner schools with groups of four students during one teaching lesson (50 min).

The following sections describe the tool for use in classrooms to fulfil the learning intentions described in Table 1 and for use as a shorter activity, e.g., during outreach events where time is limited.

## 4. Materials and Methods

The OEM system and the translational pathway were simulated in a classroom activity through: (i) assembly of the tool, (ii) production of calibration curves, (iii) taking measurements, and (iv) making a diagnosis.

**Figure 3:**
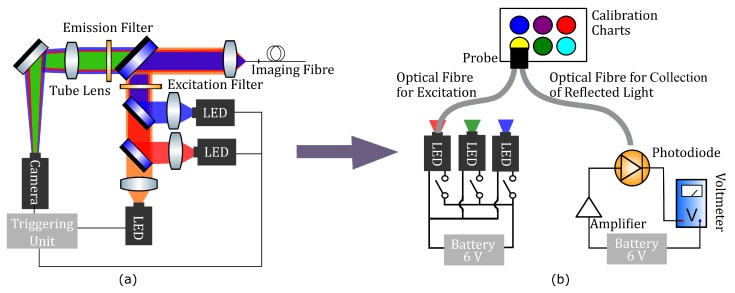
Schematics of optical instrumentation: (**a**) Schematic of the OEM system. A blue LED excites the endogenous fluorophores, especially the structural molecules of connective tissue, collagen and elastin, which emit fluorescence in the green spectral range. This autofluorescence allows clinicians to navigate through the bronchial tree and identify normal and abnormal tissue. Red and near-infrared (NIR) LEDs are used for exciting exogenous fluorophore markers of disease. (**b**) Schematic of the educational tool that can be built around an optoelectronics circuit board. Two optical fibres combine to form illumination and collection channels of a ‘sensing head’. The collection channel is directed to a photodiode/amplifier combination and voltmeter. Three LEDs are used to take measurements of reflected light from coloured patches on a calibration chart before the tool can be used on unknown samples.

The OEM system, described in detail elsewhere [73], has been translated from the lab bench to the bedside in clinic and has been deployed in first in human studies [65]. As mentioned in the previous section, the educational tool was developed to resemble the OEM system as closely as possible while being reproducible, inexpensive, and robust (Figure 3).

As depicted in Figure 3b, the tool is based on: (i) a light source, (ii) two optical fibres which combines to constitute a probe (one is used for excitation of a sample and one for collection of reflected light from the sample), (iii) a sample (calibration chart or lung), and (iv) a measuring unit with a photodiode, amplifier and voltmeter. The optoelectronics kit was comprised of an LED investigations board (26-001, JJM Electronics, Urquhart, Moray, UK) and a light sensor board (26-001, JJM Electronics, Urquhart, Moray, UK) which are commercially available (total cost: $55). The tool made use of the kit’s three (red, green, and blue) LEDs and the photodiode/amplifier combination to allow the user to interrogate the sample and make measurements. The kit was powered by a 9 V battery pack.

We note that the functions of the optoelectronics kit are straightforward and could be readily replicated with simple circuitry. A voltmeter (PG107 digital 600 V AC/DC multimeter, Precision Gold, China) was used to indicate the output of the photodiode/amplifier combination.

The probe consisted of two optical fibres and bespoke 3D printed parts that could be clipped onto the optoelectronics kit to transform the kit into a portable OEM system. The 3D printed parts were: (i) a probe tip, which holds the illumination and collection fibres at the requisite $45^{\circ }$ angle; (ii) covers, which block out errant light and optimally position the illumination and collection fibres with respect to the LEDs and photodiode/amplifier combination; and (iii) clips, which hold the two boards of the optoelectronics kits together for ease of use. Printing was done using a Bolt Pro printer (Leapfrog, Alphen aan den Rijn, The Netherlands) with a PLA/PHA (polylactic acid/polyhydroxyalkanoate filament ( 1.75 mm diameter, colorFabb, Belfeld, The Netherlands). Optical fibres have to fit the probe and cover pieces, but must not be so loose that they slip out of the pieces. Accordingly, the three main detector parts—probe piece and the LED and photodiode covers—were designed with fibre channel diameters slightly smaller than the 3 mm (± 0.05 mm) fibre width. After printing, each piece was hand-drilled using a 3.1 mm diameter drill bit, to achieve the appropriate fit. We have provided CAD design files [75] required to produce the 3D printed parts which facilitate alignment of the fibres with both the LEDs and the sample. In addition to this, a complete technical discussion of the tool, including an instructional video and troubleshooting guide, are provided in the SI.

**Figure 4:**
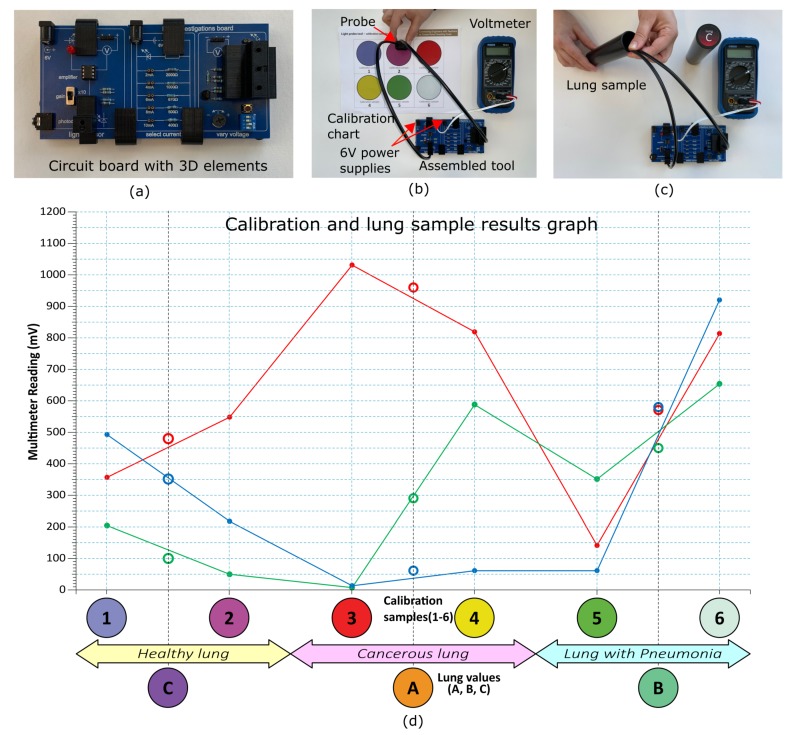
How the assembled tool is used. (**a**) The assembled optoelectronics kit. (**b**) Calibrating the tool by taking measurements of coloured patches using the optical fibres fitted into a ‘sensing head’. (**c**) Measuring the ‘lung’ samples that are coloured patches placed at the bottom of opaque tubes difficult to view directly by eye. (**d**) Example calibration curves (lines) and ‘lung’ diagnosis measurements (circles). Note that this calibration chart displays the diagnosis states of the ‘lung’ sample, which is not present on the calibration chart provided to users. Schematic diagrams of the 3D printed parts, the circuit boards, and complete setup are given in SI Figures S1–S3 of the Design, Troubleshooting, and Variations document, in addition to a full instructional video of the tool’s build and operation which can be found in [75].

For the two optical fibres, 3 mm bare polymer fibres OMPF3000, OMC, Redruth, UK) were purchased in 5 m reels, and cut to 50 cm lengths. Each fibre end was cut perpendicular to the fibre direction, and the ends polished to a fine finish using a two grade perspex polishing kit. The fibres were then sheathed in 4 mm black plastic sleeves (PVC-4-0-CL, Pro-Power, China), see Figure 4b.

The samples consisted of either a calibration chart or a phantom lung. The calibration chart was printed on an A4 sheet of paper (see the following section and SI for more details) while the lung was represented by a long tube inside of which was a colour patch which could not be seen directly by eye, see Figure 4c. The tubes were constructed from standard 32 mm diameter black ABS (acrylonitrile butadiene styrene) tubing (available from hardware stores), each 3 m length cut into 15 cm lengths. 32 mm discs were punched from 3 mm ABS sheeting, and plastic-welded to the base of each tube, after the appropriate ‘lung’ sample colour disc, printed on 160 gsm printer paper, was fixed to the inside surface. The tubes are too deep for the coloured patches to be seen directly by the eye. To prevent spurious reflections inside the ABS tube from disrupting measurements, rectangles of matte black art paper were cut, curved and glued to the inside of each tube.

## 5. Demonstrating the Use of the OEM Educational Tool

### 5.1. Using the Education Tool in a Classroom Setting

With this educational tool, two main concepts are introduced: (i) optical fibres can be used as passive sensors for collection and transportation of light/information; (ii) changes in environment, here colour, can be interrogated with light and detected as a varying voltage reading. Additionally, the students learn the preparation of calibration curves with the option of extending this into a teaching module about uncertainties and misdiagnosis.

For calibration of the tool, light from each of the LEDs is coupled into one of two optical fibres and guided to the 3D printed ‘sensing head’ which is brought in direct contact with coloured patches on a calibration chart (Figure 4b). The reflected light is coupled into the second optical fibre which is mounted at a $45^{\circ }$ angle to maximise collection efficiency whilst minimising coupling with errant LED light. The collected light is guided to a photodiode/amplifier combination connected to a voltmeter allowing a voltage to be recorded. The calibration process uses a set of six known coloured patches, from each of which a set of three readings are produced and graphically plotted. An example of the readings from six coloured patches (noted as 1 to 6 in the *x*-axis) is provided in Figure 4d for the red, green and blue light. To account for the possible range of voltage readings expected as a result of variations in components, for example battery power or optical fibre transmission characteristics, the calibration graph features two *y*-axes spanning different ranges (0mV–600mV and 0mV–1200mV). Measured calibration values are sufficiently different for each sample to accommodate broad errors in operation of the tool and in variations in supplied power, while still producing an acceptable calibration graph. Calibration graphs can be found in the Supplementary Materials [75].

The actual measurements are made from three ‘lung’ samples; long tubes containing coloured patches that cannot been seen directly by eye. Each ‘lung’ sample colour is chosen to lie between two of the calibration colours, and can be plotted against the calibration graph, allowing students to realise their own ‘diagnosis’ of the ‘lung’ (Figure 4). ‘Lung’ A produces a reading between calibration samples three and four and leads to a diagnosis of cancer; ‘lung’ B produces a reading between calibration samples five and six and leads to a diagnosis of pneumonia; and ‘lung’ C produces a reading between calibration samples one and two and represents a healthy lung. A video explaining the whole process, from assembly to measurement in the ‘lung’ samples as well as complete setup and operation instructions are provided in [75].

### 5.2. Using the Educational Tool in Shorter Engagement Activities

The same hardware that was developed for use in classrooms was also used as the basis of a 2-min challenge for use at science festivals with a wider audience. In that context, several steps are removed—notably the calibration of the tool and all of the classroom-focused teaching materials. Instead, a calibration chart was pre-filled prior to the event (similar to the one in Figure 4d). Each line of the chart gives access to a unique colour combination—corresponding to a voltage reading using red, green and blue light characteristic of an infection caused by a specific pathogen. The exercise consisted of diagnosing a single ‘lung’ by recording voltage values using each of the three LEDs, and then comparing the observed values to the calibration table pre-recorded data. Identification of the causative pathogen would then help participants identify the most appropriate treatment to cure the patient. During the activity, pills of different colours were made available and only pills corresponding to the colour of the ‘lung’ sample would be beneficial to the patient. At the end of the 2-min challenge, participants were shown the colour patch hidden inside the ‘lung’ sample for comparison with their recommended diagnosis. By simply taking the three measurements and looking up what they related to, participants could get a feel for the research without the deeper understanding that takes multiple lessons to achieve.

## 6. Conclusions

A relatable context is frequently key to producing truly engaging activities for schools, festivals and museums. We have shown an affordable and robust tool that can be used in a school setting as well as being easily simplified to a configuration suitable for demonstration at a science festival or other public engagement events. Our tool can be used to demonstrate interdisciplinary research along with providing an engaging context for the study of light and fibre optic systems. We propose that teachers represent an often-overlooked resource for researchers to improve the quality and relatability of their public engagement activities, and that, by engaging directly with such professionals, we have seen improved engagement of students with state-of-the-art research in the classroom. The key to such collaborative work is that teachers and researchers should work together from the outset, rather than only allowing post-hoc teacher approval of activities produced by researchers. This novel teaching activity, which supplements secondary science education, introduces clinical challenges and pathways through the use of a case study and has been successfully deployed in 10% of Scottish secondary schools. The process, from development to testing of the sensors in schools, was evaluated by an independent evaluator. Data gathered were from a rich range of sources at various points throughout the project, including pre- and post-project survey, informal discussion, observation field-notes, etc. In total, sensors were produced for 30 teachers from Scottish secondary schools and 60 students participated in a pilot study. In addition, 100% of teachers and students reported the project as enjoyable, interesting and informative. Furthermore, 100% of teachers who received training to use the tools plan to use them again and 93% of students said the tools were interactive and absorbing. In addition, 70% of students who took part in the study thought that engineering/research was not enjoyable or interesting before the session. After the session with the tool, 100% thought that engineering could be enjoyable or interesting. We intend to carry out a follow-up study to further quantify the longer-term impact of this tool in schools.

## Figures and Tables

**Table 1:** Learning intentions and core tasks. Identification of curriculum areas from the experiences and outcome document for sciences which were most clearly linked to the research and the core tasks that address these. Key: OEM—optical endomicroscopy.

Learning Intentions	Link from Curriculum to Research	Core Task
Understanding of organ systems	The impetus for the OEM system modelled is to tackle the lung and its diseases	Engagement with appropriately targeted teaching materials, aimed at student-age readers and providing guidance for teacher-led or peer investigation of these curriculum topics
Researching new developments	The OEM system modelled by the educational tool is an ongoing piece of work with regular production of peer-reviewed publications [30,72,73]		
Light	The construction and understanding of the educational tool requires knowledge of optical fibres to get light to and from inaccessible spaces, colour theory to interpret what occurs at the distal end, and the use of a number of circuit components to produce a working measurement system	Use of the educational tool itself in classroom environment. The focus on calibration and the reduction of errors facilitates the passive teaching of basic scientific literacy
Optical fibres		
Colour mixing		
Building a circuit		

## Supplementary Materials

The following are available online at https://www.mdpi.com/1424-8220/20/2/402/s1.
