# Supplementary material for: Demonstrating the Use of Optical Fibres in Biomedical Sensing: A Collaborative Approach for Engagement and Education
Source: Sensors (Basel). 2020 Jan 10;20(2):402. doi: 10.3390/s20020402 (PMC7014119; doi:10.3390/s20020402)
Supplement: Supplementary file 1 [file sensors-20-00402-s001.pdf]

Supplementary Information

Demonstrating the use of optical fibres in biomedical sciences: a collaborative approach for engagement and education

Katjana Ehrlich[1,9,+], Helen E. Parker[1,10,+], Duncan K. McNicholl[1,2], Peter Reid[3], Mark Reynolds[3], Vincent Bussiere[4], Graham Crawford[5], Angela Deighan[6], Alice Garrett[4], Andras Kufcsák[1], Dominic R. Norberg[1], Giulia Spennati[4], Gregor Steele[7], Helen Szoor-McElhinney[1], and Melanie Jimenez[4]

[1]EPSRC IRC Hub in Optical Molecular Sensing & Imaging, Centre for Inflammation Research, Queen's Medical Research Institute, University of Edinburgh, Edinburgh, EH16 4TJ, UK
[2]Scottish Universities Physics Alliance (SUPA), Institute of Photonics and Quantum Science, Heriot-Watt University, Edinburgh EH14 4AS, UK
[3]College of Science and Engineering Engagement Team, King's Buildings, University of Edinburgh, Edinburgh EH9 3BF, UK
[4]School of Engineering, Biomedical Engineering Division, University of Glasgow, Glasgow G12 8LT, UK
[5]Liberton High School, Edinburgh EH17 7PT, UK
[6]St. Margaret Mary's School, Glasgow G45 9NJ, UK
[7]Scottish Schools Education Research Centre, Dunfermline KY11 8UU, UK
[9]Katjana.Ehrlich@ed.ac.uk
[10]H.Parker@ed.ac.uk
[+]these authors contributed equally to this work

This file includes:

- Supplementary text on the following:
    1. Design of the optical endomicroscopy (OEM) tool
    2. Setting and troubleshooting up the tool
    3. Calibrating the tool
    4. Variations of components
    5. Teaching aims
- Supplementary figures:
    - SI-Fig. 1 to SI-Fig, 8

1 Design of the optical endomicroscopy tool (OEM) tool

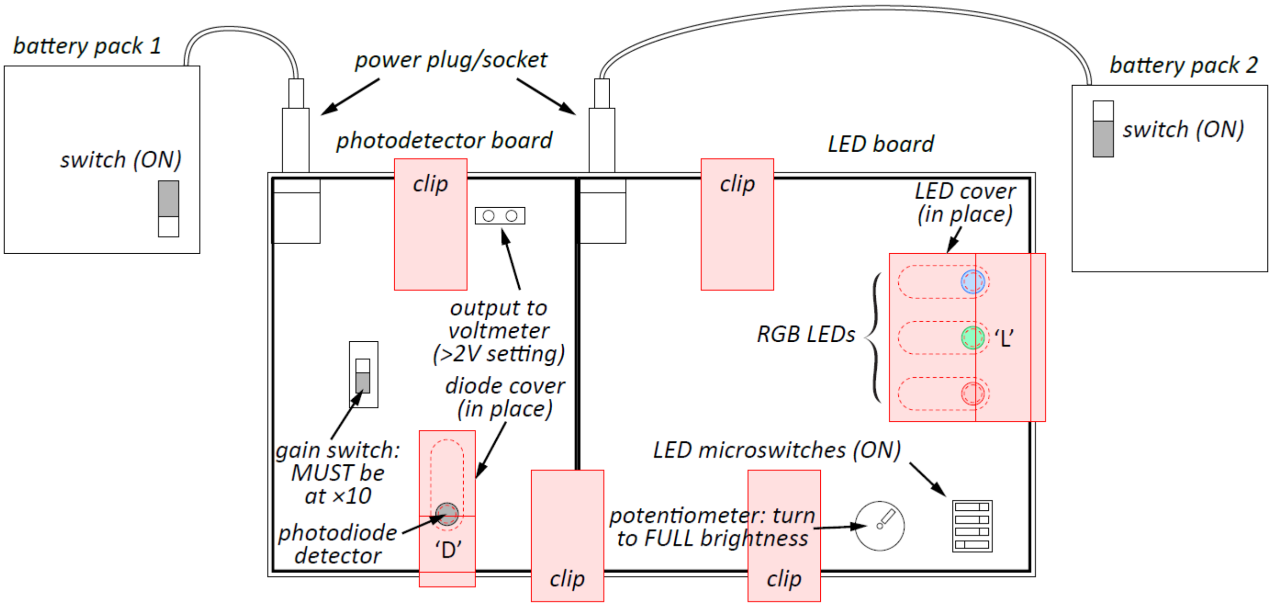

SI-Fig. 1. Schematic of assembled OEM tool making use of bespoke 3D-printed parts and an existing commercial optoelectronics kit.

Optical endomicroscopy (OEM) uses a single, thin fibre to transmit light outwith the visible spectrum, which then activates fluorescent compounds placed on the lung; this activated light is then returned to a detector, where analysis of the spectrum allows a medical diagnosis to be made. Under the constraints described in the manuscript, while still reflecting the operating principles of a real OEM system, the OEM tool had to be designed to work in a classroom environment, and the following changes were made.

- Thin fibre optics require either an extremely bright (and hot) light source or a highly sensitive detector. The former imposes cost and safety constraints, and the latter requires a bespoke circuit design: both were unsuitable for schools, so a 3 mm optical fibre was chosen, which had the added advantage of being considerably stronger than the standard thin fibres
- For simplicity, a dual fibre approach was chosen: one fibre to supply light to the sample and the other to transmit the reflected light to the detector

The OEM tool makes use of reflected light from coloured patches embedded in 'lung' samples, which are designed to be difficult to view directly. Each of the red, green and blue LEDs in the optoelectronics kit is in turn directed by optical fibre onto the samples, and the reflected light components are measured by the photodiode/amplifier combination, producing a series of triple-valued results for each coloured patch. Many Scottish secondary schools make use of an existing commercial optoelectronics kit, comprising a three-LED (R,G,B) source board, and a basic photodiode/amplifier combination. The advantages of using such boards were that:

- New kit did not have to be purchased
- The existing kit was well understood by schools
- The kits were already used in experiments that form part of the science curriculum, and were thus a stepping stone to the more advanced light tool activities

Tests showed that the LEDs, in combination with the 3 mm optical fibre, were sufficiently bright to reflect light onto the photodiode, and a reasonable range of voltage readings could be made from the amplifier. To complete the light tool, 3D-printed components were designed to accurately place the optical fibres against the LEDs and photodiode, and to allow light within a 'probe' piece to reflect from the test sample to the detecting fibre.

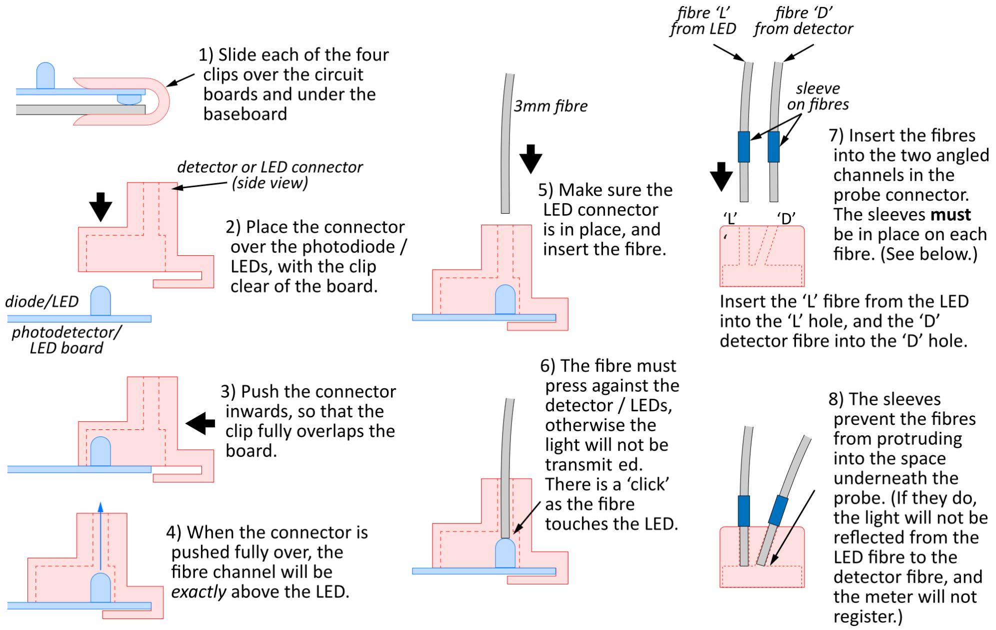

SI-Fig. 2. How the 3D printed parts should be used in the assembled tool.

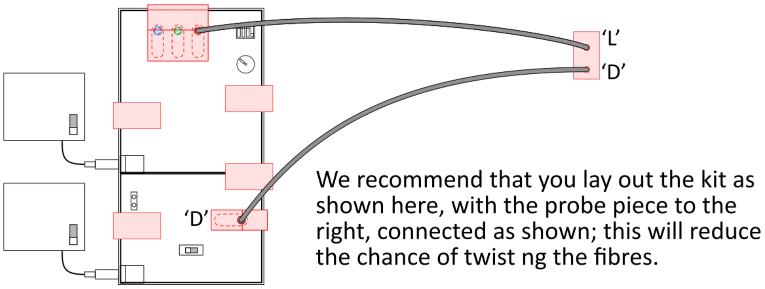

SI-Fig. 3. Assembled tool with fibre probe and sensing head.

2 Setting up and troubleshooting

Set up the device as shown in (SI-Fig. 1, SI-Fig. 2 and SI-Fig. 3). With both battery packs on, use calibration samples one and four to check the setup, these samples are shown in (SI-Fig. 4). The voltmeter readings will be between 0.1 V and 3 V: if everything is in order, for sample one you should get a high reading (> 1.5 V) for red, and small (~ 0.2 V) for blue and green. Sample four will give a high (~ 0.8 V) blue reading, with lower red and green. If there is a low (or no) signal on the voltmeter, check the following:

1. The battery packs are both ON
2. The battery packs are putting out 6 V
3. The gain for the amplifier on the photodetector board is set to '$\times$ 10'
4. The potentiometer on the LED board is at full, and the LEDs are as bright as possible
5. The fibres to the detector and LEDs are firmly in contact; if the connectors are not fully in place the fibres will not be above the LEDs
6. The 'L' and 'D' fibres are inserted into the correct hole in the probe
7. The fibres in the probe are not protruding into the space under the probe. If they are the light will not be properly reflected and the signal will be much reduced

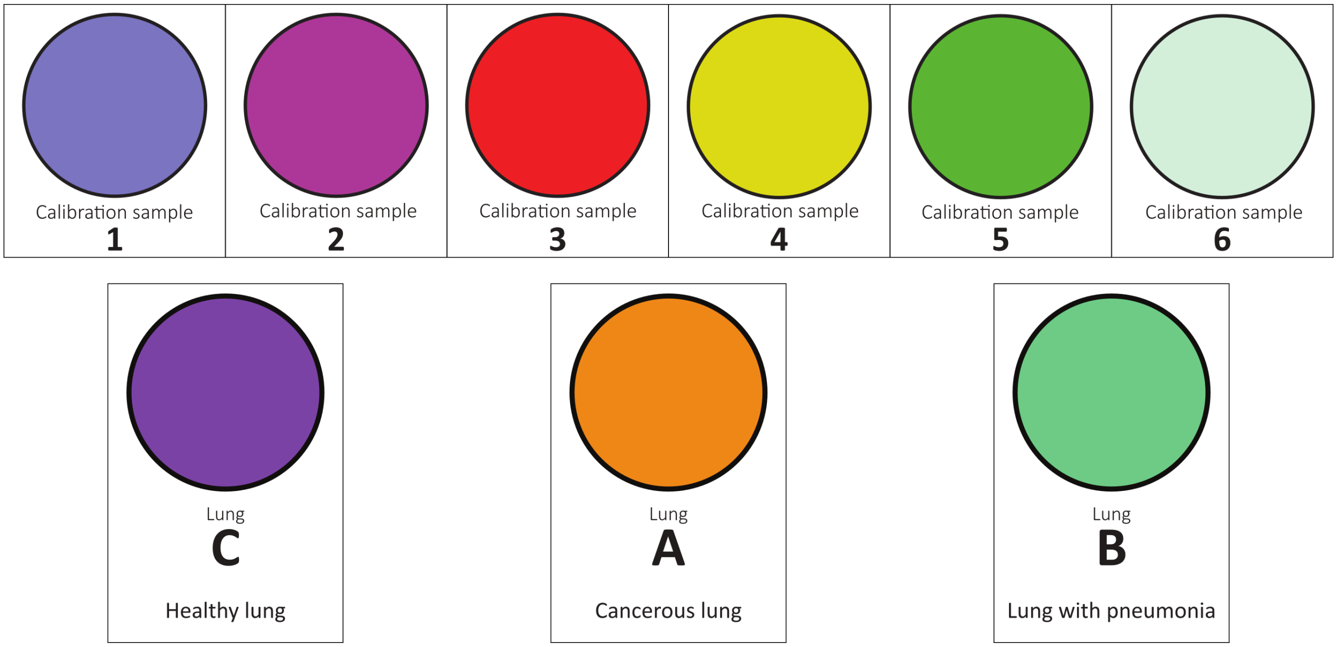

SI-Fig. 4. Calibration samples (1 - 6) for OEM tool. 'Lungs' A - C were chosen to lie in between the calibration samples. The coloured 'lung' patches are set within opaque tubes so that they are not visible to the eye.

3 Calibrating the tool

The tool is calibrated by using an existing set of known coloured patches (SI-Fig. 4) from which readings are recorded into a table (SI-Fig. 5) and three graphs plotted (SI-Fig. 6), one for each LED. The colour chart for calibration and the 'lung' patch colours were chosen to satisfy the following requirements:

- Each sample produces a distinct set of red, green and blue reflectance values, different from all other calibration samples.
- The values are sufficiently different for each calibration sample to accommodate broad errors in operation of the tool and in variations in supplied power, while still producing an acceptable calibration graph.
- The values were also chosen to permit some variation in the printing of the colour sample charts, should schools need to produce new copies. The 'lung' sample colours are fixed in this version of the tool, and a poorly aligned colour printer might produce a set of calibration colours whose graph would not then fit the 'lung' measurements. We carried out extensive testing using a variety of printers, and in all cases the resultant graphs still allowed correct 'lung' values to be taken; this remains a potential problem, however.
- The three 'lung' sample colours were chosen to lie at intermediate points between the calibration colours: each of the red, green and blue components were fixed to lie midway between the components of the two adjacent calibration colours.
- 'Lung' A was chosen to have a set of component values that were easy to place, even with poor measurement or operational technique. 'Lungs' B and C were chosen (more accurately, the adjacent calibration samples were tweaked) such that less careful measurement might lead experimenters to misidentify the samples. The diagnoses for these samples were deliberately chosen such that sample C was healthy, while B represented a patient with pneumonia. This allows teachers to discuss the real-world consequences of medical misdiagnosis: false-positives and false-negatives could arise.

Having constructed the calibration graph, the pupil experimenters then measure the colour readings from three 'lung' samples: long tubes into which coloured patches have been placed, too deep to be seen directly by eye. Each 'lung' sample colour is chosen to lie between two of the calibration colours, and so can be plotted against the calibration graph, allowing a final diagnosis of the health of the 'lung' to be made. (SI-Fig. 6) shows a completed calibration graph, against which 'lung' sample readings have been placed. Reading of the 'lung' samples can be aided by an acetate sheet (SI-Fig. 7) as shown in Supplementary Video 1.

The light tool was designed to cope with inevitable variations in tool components. The optical fibres vary widely in their transmission and collection characteristics depending on the quality of the fibre end face. In the optoelectronics kits, the

Calibration sample	Multimeter reading (V) to two decimal places (eg. 1.21) Red LED	Green LED	Blue LED
1			
2			
3			
4			
5			
6			

Lung			
A			
B			
C			

SI-Fig. 5. Results table of voltage readings from each of the three LEDs (RGB) using the six calibration patches from the calibration chart and the three 'lung' samples.

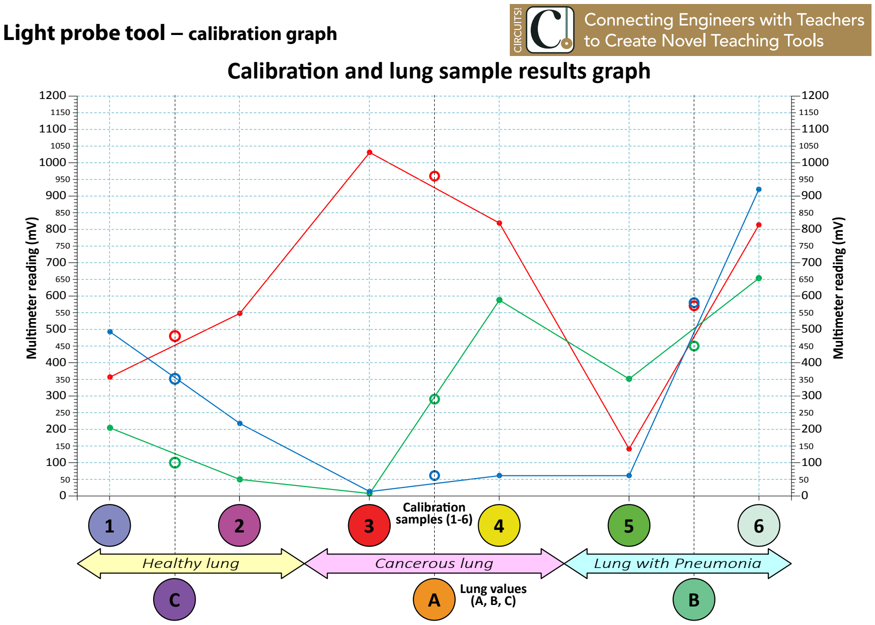

SI-Fig. 6. Example of a completed calibration graph that can be used to make a diagnosis of the 'lung' condition.

sensitivity of the amplifiers varies across kits and the LED characteristics change with age and usage. We found that for some LEDs a 0 mV to 600 mV axis range was suitable and for some a 0 mV to 1200 mV axis range was more appropriate. To determine the suitable axis range it is advised that the red patch be measured under red LED illumination first as this gives the highest voltage reading of all patch/LED permutations.

The optoelectronics kits can be powered either by power plugs or by battery packs. The battery packs supply a nominal 6 V DC but the actual output can vary substantially from this value. While the amplifier and photodiode are relatively insensitive to the supply voltage, and operate at voltages as low as 4 V, the brightness of the LEDs drops considerably and the decrease varies across the LEDs. (SI-Fig. 8) shows the results of tests carried out between 4 V to 6.5 V, below 4 V the amplifiers no longer operate. As can be seen from the figure, while the vertical voltage span for the calibration and 'lung' measurements shrinks considerably as supply voltage is reduced, it is still possible to use the tool, and to derive correct 'lung'

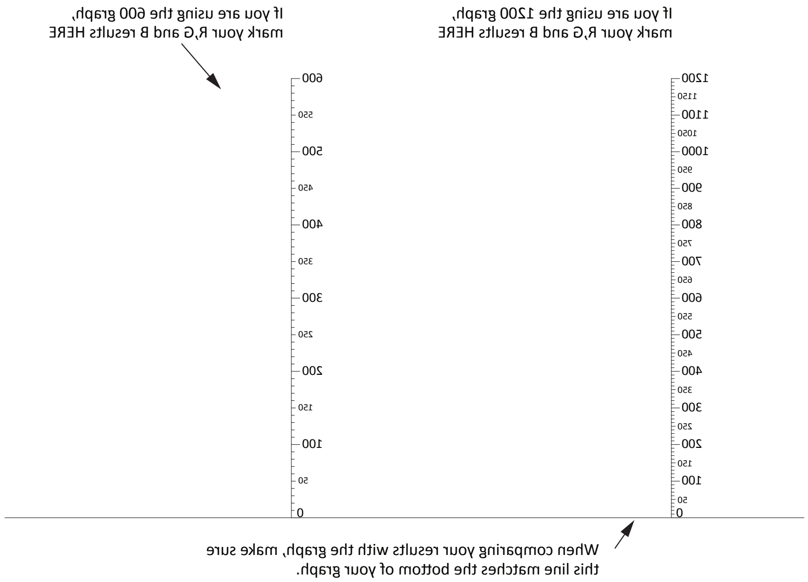

SI-Fig. 7. Acetate sheet for reading the ‘lung’ results. Students can mark on their triple value ‘lung’ readings using a drywipe marker and can slide the acetate along their calibration graph in order to find the best match. This process can be seen in Supplementary Video 1.

values on the calibration graph, though the possibility of misdiagnosis does rise. To ensure that battery pack voltage levels or other manufacturer variations do not affect the ‘lung’ diagnosis, we recommend production of the calibration graphs and measurements on the same kit and within one lesson.

The paper quality and printing quality of the colour patches also greatly influence the voltage readings. Here, we used coloured patches with CMYK values which on a monitor screen were close to RGB values, within the chosen CMYK gamut. For reasons of simplicity and economic efficiency, we chose the concept of reflection. If the concept of fluorescence were to be maintained, we recommend Autofluorescent Plastic Slides (92001, $20, Chroma).

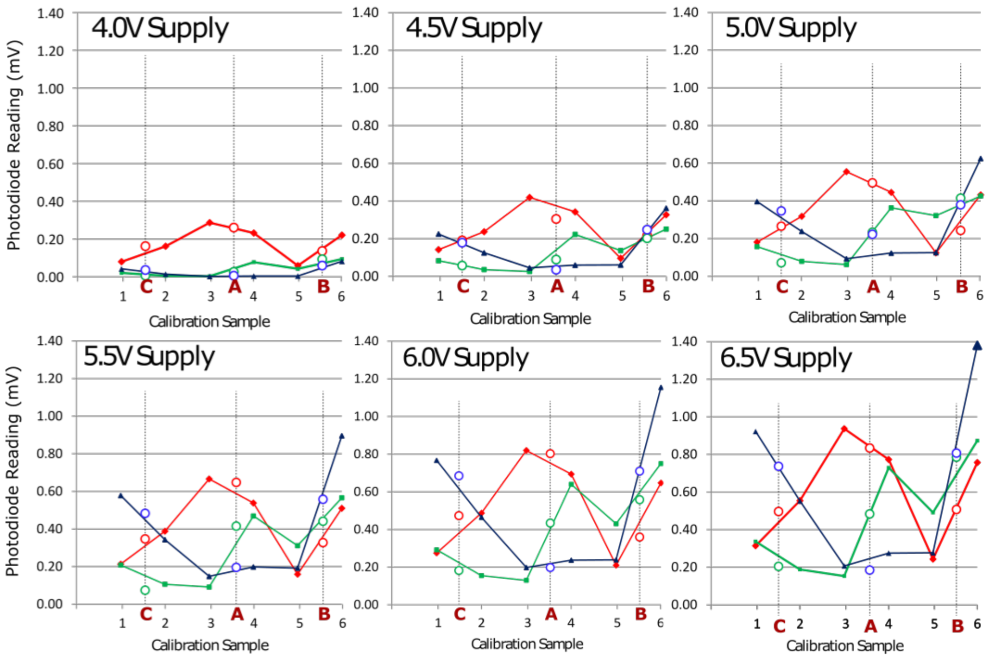

SI-Fig. 8. Calibration variation as a result of changing supply voltage in the range of 4 V to 6.5 V. Significant reduction in the photo-multiplier reading can be seen as the supply voltage drops, although the tool can still be used to infer a diagnosis within this supply voltage range.
